# Assessment of a new class of slow γ-aminobutyric acid type A receptor anesthetic on mouse neuronal and astrocyte mitochondrial function

**DOI:** 10.21203/rs.3.rs-6659152/v1

**Published:** 2025-09-11

**Authors:** Creed M. Stary, Brian Griffiths, Xiaoyun Sun, Frances Davies, Alam Jahangir, Elizabeth Manis, Isabella Russo, Claire Ai Jue Dean, Lillen Montague, Edward Bertaccini

**Affiliations:** Stanford University School of Medicine; Stanford University School of Medicine; Stanford University School of Medicine; VA Palo Alto Health Care System; VA Palo Alto Health Care System; Stanford University School of Medicine; Stanford University School of Medicine; Stanford University School of Medicine; Stanford University School of Medicine; VA Palo Alto Health Care System

**Keywords:** GABAAR, GABA, oxygen consumption, bioenergetics, oxidative phosphorylation, reactive oxygen species, mitochondrial membrane potential, brain

## Abstract

Our previous work has demonstrated efficacy of a new chemical class of the slow γ-aminobutyric acid type A receptor anesthetics that produce minimal effects on breathing and hemodynamics in rats. To advance pre-clinical testing, we screened one member of our class of compounds, KSEB 14 – 01, for mitochondrial toxicity in primary neuronal and astrocyte cultures from mice. Prior to treatment, cell cultures were incubated with: Mitotracker GreenTM to assess mitochondrial density, tetramethylrhodamine ethyl ester to assess mitochondrial membrane potential, dihydroethidium to assess reactive oxygen species (ROS), and 4′,6-diamidino-2-phenylindole for cell counting. Cultures were treated with either propofol or KSEB 14 – 01 (dissolved in dimethylsulfoxide) at concentrations of 5, 10, or 30μM. ROS and mitochondrial membrane potential were measured for 6h after which mitochondrial density and cell proliferation were quantified. In parallel experiments, continuous oxygen consumption rates (OCR) were measured during glucose deprivation (GD) in astrocyte cultures. No significant differences were observed in cell count or between treatments. In both neurons and astrocytes, mitochondrial membrane potential was decreased at 6h with 5μM propofol and KSEB 14 – 01 treatment, but remained stable with higher doses of propofol and KSEB 14 – 01. Neuronal ROS generation increased in all groups by 6h but was significantly lower with 10 and 30μM propofol and KSEB 14 – 01. In all treatment groups, astrocyte ROS generation was significantly increased after 6h, with no differences between groups. After 48h GD astrocyte OCR was maintained with all doses of KSEB 14 – 01 and with the highest dose of propofol. In conclusion, this study demonstrates that KSEB 14 – 01 does not exhibit any overt mitochondrial toxicity relative to propofol in either neurons or astrocytes. Future in vivo work is warranted to explore the mechanisms and applications for maintenance of bioenergetic equivalents during cell stress with KSEB 14 – 01.

## INTRODUCTION

Intravenous (IV) anesthetic agents currently in clinical use today share the common pitfall of inducing hemodynamic suppression at therapeutic doses. While hypotension can typically be effectively countered in the controlled environment of the operating room, in remote settings, the preferred trauma anesthetic is typically ketamine, which is not cardio-depressive. However, ketamine is associated with a host of unwanted side effects including extreme delirium, excessive salivation, and increased intracranial and intraocular pressure. For the above reasons, our prior work [1] focused on identifying novel IV anesthetics with improved safety and efficacy profiles versus existing anesthetics, whether in the operating room, ambulance, or trauma center. To achieve this, we employed a novel 3D computational model that enabled high-throughput in silico screening of new molecules that are highly selective for the slow γ-aminobutyric acid type A receptor (GABAAR). Our novel class of GABAAR-targeted “KSEB” anesthetics demonstrates comparable anesthetic efficacy as currently available IV anesthetics, but without the constraints of any cardio-depressive effects.

One of the proposed mechanisms for hemodynamic depression with IV anesthetics is modulation of mitochondrial function [2–4]. Adenosine triphosphate (ATP) is the main energy currency of cells, and any reductions in intracellular ATP availability relative to demand can depress cellular function, including both cardiac contractility and neuronal activity. Oxidative phosphorylation occurs within the mitochondria via the electron transport chain (ETC) to provide the principal source of ATP for most cell types and cellular processes. However, mitochondria are highly vulnerable to bioenergetic uncoupling, disrupting ETC efficacy and ultimately reducing stoichiometric ATP availability to match demand. Commonly given anesthetics can interact with mitochondria and affect their structure or impair respiratory chain functioning with decreased ATP production [2–5]. In this context, mitochondria have been considered as the potential site of action of general and local anesthetics [2]. However, whether our newly developed KSEB GABAAR-targeted anesthetics modulate mitochondrial function has not been investigated. Therefore, in the present study, we assessed the effect of an isolated KSEB compound on mitochondrial membrane potential, ROS production, and oxygen consumption rates in primary brain cell cultures at rest and in response to cell stress.

## METHODS

### Generation and validation of KSEB 14–01

**Figure 1:**
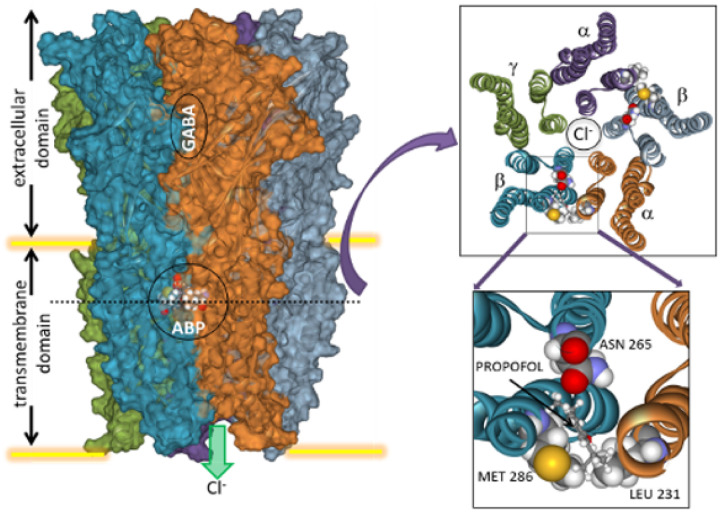
*In silico*model of the anesthetic binding site within the slow γ-aminobutyric acid (GABA) type A receptorlocated within the outer third of the transmembrane domain between alpha helical bundles and bounded by 3 key amino acids. APB: anesthesia biding pocket.

**Figure 2:**
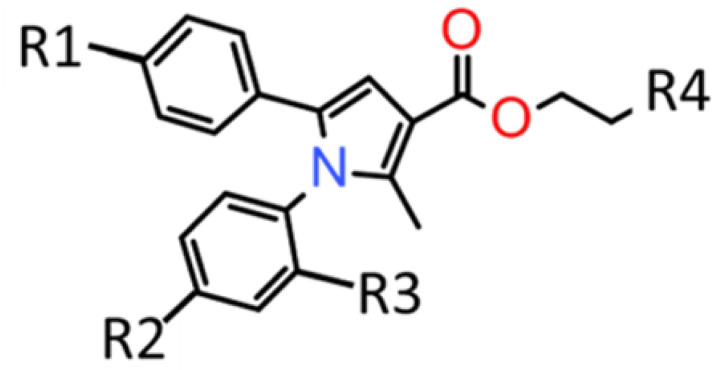
Molecular structure of the KSEB class of GABA_A_R agonists. For KSEB 14–01 R1=Cl, R2=Cl, R3=Cl,

We used predictive modeling to identify over 100 derivative structures and docked them to our GABAAR model (Fig. 1) to assess for potency as previously [1]. Compounds with verified structures via NMR, purities consistently >90%, and demonstrating minimal in silico toxicity predictions compared to propofol were selected for additional testing [1]. Figure 2 demonstrates the structure of the KSEB class of GABAAR small molecule agonists. The top 30 were then tested for anesthetic potency using tadpole loss of righting reflex (LORR, [1]). KSEB 14–01 demonstrated an ED50 = 0.28uM for tadpole LORR and was selected for further testing. To determine the safety and selectivity of our compounds for the GABAAR KSEB 14–01 to the SAFETYscan47 E/IC50 ELECT battery of tests (Eurofins) at a dose range of 2 nM to 50 mM through a 47 human receptor assay panel.

### Primary brain cell cultures and KSEB treatment

All experiments using animals were performed according to protocols approved by the Stanford University Animal Care and Use Committee and in accordance with the National Institutes of Health guide for the care and use of laboratory animals. Primary cortical astrocytes were prepared from postnatal (days 1–3) mouse pups from pregnant dams (Charles River) as we previously described [6]. Briefly, following euthanasia with isoflurane, neonatal brains were isolated, and cortices were microdissected and dissociated with 0.05% trypsin (ThermoFisher Scientific) in DMEM medium (ThermoFisher Scientific) for 20 min. Following trypsinization, cells were dissociated by trituration and then resuspended in DMEM medium supplemented with 10% FBS and 1% penicillin/streptomycin and plated in 24-well cell culture plates. The culture medium was exchanged every two days, and the cells were used for experiments after >70% confluence at 16–18 days in vitro (DIV). Primary neuronal cultures were prepared as we previously described [6] from embryonic (E16-E18) mouse cortices. Briefly, the dissected cortices were dissociated with 0.05% trypsin and EDTA for 15 min at 37 °C, triturated, then plated in medium containing 5% ES and 5% FBS (HyClone). A relatively pure neuronal culture was obtained by adding Cytosine arabinoside (3 mol/liter, Sigma) 24h after plating to curb glial proliferation. Cultures were treated with: plain culture media; 1:5000 dimethyl sulfoxide (DMSO, Sigma Chemicals); propofol (in 1:5000 DMSO, Sigma Chemicals) at final concentrations of 5, 10, or 30mM; or KSEB 14–01 (in 1:5000 DMSO) at final concentrations of 5, 10, or 30mM.

### Live-cell fluorescent imaging

Living primary astrocyte and neuronal cultures were imaged using an automated LumaScopeTM 720 (Etaluma) with a 20X fluor objective as we previously reported [7]. Cells were maintained at a temperature of 37 °C and a concentration of 5% CO2 in an atmospherically controlled imaging chamber (Ibidi GmbH) for imaging of live-cell intracellular kinetics [8]. For assessment of mitochondrial membrane potential, astrocytes were incubated for 30min with tetramethylrhodamine ethyl ester (TMRE, 50nM, ThermoFisher Scientific) at 37°C, and the same concentration of TMRE was maintained in all bathing solutions throughout the experiments [8]. Oxygen radical production was monitored as we previously reported [8] with hydroethidine (HEt, ThermoFisher Scientific). Cultures were incubated in the dark with 5mM HEt in BSS5.5 (30min, 37°C), and the same concentration of HEt was maintained in the bath throughout each experiment. Automated unbiased mean intensity of fluorescence was measured Image-J v1.49b software (NIH) with fixed threshold parameters for all treatments [8]. Changes in fluorescence were normalized to the basal fluorescence for each cell at the start of the experiment [8]. In parallel, cultures were incubated for 30 mins at 37 °C and 5% CO2 with Mitotracker GreenTM (5mM) to assess mitochondrial density, and with the nuclear dye DAPI (4′,6-diamidino-2-phenylindole, ThermoFisher Scientific) to standardize fluorescence to total cell number [8].

### Measures of oxygen consumption rates during substrate limitation

Nunc flat-bottom 96-well microplates (Thermofisher Scientific) were pre-coated with poly-D-lysine (Invitrogen) for 2h before primary mouse astrocytes were seeded at a density of 1.8 hemispheres/10ml medium at a total volume of 120 mL. Oxygen consumption rates (OCR) and oxygen concentration were continuously measured using the ResipherTM oximeter (Lucid Scientific) at 37 °C, 10% CO2 over 72 hours [9]. The Resipher oxygen sensing lid contains microprobes with optical oxygen sensors that continuously scan between 0.55–0.95 mm above the cells to measure the oxygen concentration gradient within the medium column to determine OCR [9]. Baseline OCR and oxygen concentrations under 100 μL medium were measured for 6h prior to substrate limitation, and then continuously for the remainder of the 72h protocol. Glucose deprivation (GD) injury was selected as an ischemia-like stress for astrocyte cultures and was performed as we have described previously [10]. Briefly, cells were washed twice with medium lacking glucose, separated by a 15-minute equilibration period. Control-treated cells received two washes of normal medium.

### Statistical analyses

All data were expressed as mean ± SEM; the number of animals and samples is represented in figure legends. For analyses of single time points, a one-way ANOVA was performed, followed by Dunn’s multiple comparisons test for significance. For analyses of changes over time, a two-way ANOVA with repeated measures was performed, followed by Dunn’s multiple comparisons test for significance. Statistical analysis of all data was performed by GraphPad Prism 8.1.1 (GraphPad Software, San Diego, California, USA, www.graphpad.com), and P < 0.05 was considered statistically significant. All in vitro experiments were performed in triplicate, represented by at least three separate dissections.

## RESULTS

The results of the safety and selectivity testing demonstrated that KSEB 14–01 was selective for GABAAR receptor potentiation with an RC50 of 1.73mM, with no significant toxicity reported. No differences were observed in cell proliferation or mitochondrial density between treatments in either neuronal or astrocyte cultures.

### Mitochondrial membrane potential

**Figure 3:**
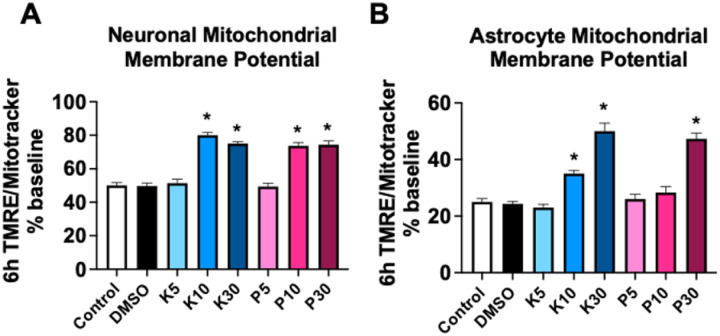
Mitochondrial membrane potential in primary mouse neuronal cultures **(A)** and in primary mouse astrocyte cultures **(B)** 6h after incubation with either untreated media, DMSO or 5mM, 10mM or 30mM propofol or KSEB 14–01. Mean±SEM; *p<0.05 versus untreated control; graphs represent 3 independent experiments.

In both primary neuronal and astrocyte cultures, mitochondrial membrane potential was significantly (p<0.05) decreased at 6h in both the untreated media and DMSO groups (Fig. 3A). This effect was likely due to phototoxicity from repeated fluorescent measures (Fig. S1). A similar effect was observed in neuronal cultures with 5mM propofol and KSEB 14–01 treatment. However mitochondrial membrane potential in neuronal cultures remained stable with 10mM and 30mM of both propofol and KSEB 14–01 (Fig. 3A). In astrocyte cultures mitochondrial membrane potential was significantly (p<0.05) disrupted after 6h incubation with 5mM propofol, 10mM propofol and 5mM KSEB 14–01, however mitochondrial membrane potential was preserved with 30mM propofol and both 10mM and 30mM KSEB 14–01 (Fig. 3B). These results identify a mitochondrial protective effect with both propofol and KSEB 14–01.

### Reactive oxygen species generation

**Figure 4:**
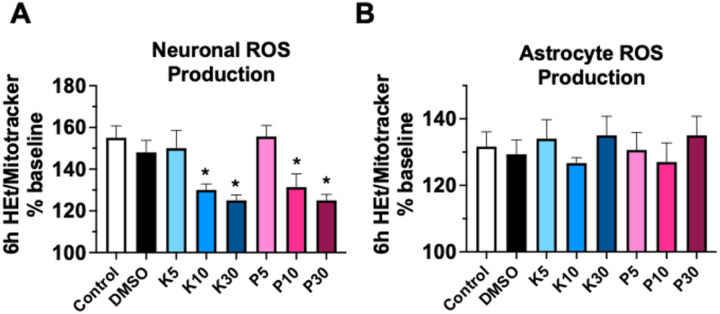
Generation of reactive oxygen species (ROS) in primary mouse neuronal cultures **(A)** and in primary mouse astrocyte cultures **(B)** 6h after incubation with either untreated media, DMSO or 5mM, 10mM or 30mM propofol or KSEB 14–01. Mean±SEM; *p<0.05 versus untreated control; graphs represent 3 independent experiments.

Neuronal ROS generation increased in untreated media and DMSO groups by 6h, and with 5mM propofol and KSEB 14–01 treatment (Fig. 4A). This was similarly an effect secondary to phototoxicity from repeated fluorescent measures. However, neuronal ROS production was significantly decreased with 10 and 30mM propofol, and with 10 and 30mM KSEB 14–01 treatment (Fig. 4A). In astrocytes, the effect was different. Contrary to the protective effect observed with mitochondrial membrane potential with higher doses of either 10 and 30mM propofol, or 10 and 30mM KSEB 14–01, no decrease in ROS production was observed with any dose (Fig. 4B).

### Oxygen consumption rate

**Figure 5:**
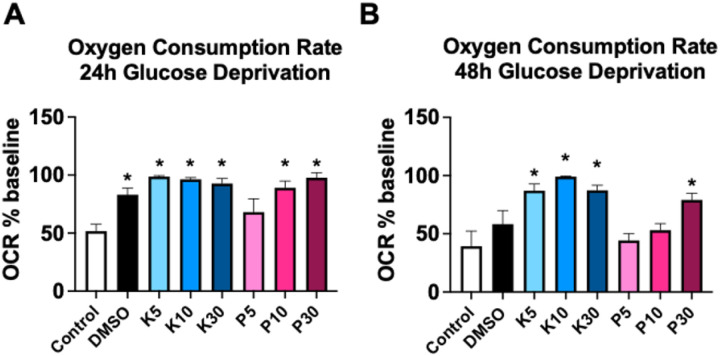
Relative change in oxygen consumption rate (OCR) in primary mouse astrocyte cultures incubated with either untreated media, DMSO or 5mM, 10mM or 30mM propofol or KSEB 14–01 after either 24h **(A)** or 48h **(B)** of glucose deprivation (GD) injury. Mean±SEM; *p<0.05 versus untreated control; graphs represent 3 independent experiments.

Prior to GD, no alterations were observed in OCR during the 6h equilibration period (Fig. S2). After 24h of GD, there was a significant (p<0.05) decrease in OCR only in astrocytes in the untreated media group and with 5mM propofol (Fig. 5A). After 48h of GD OCR remained preserved in astrocytes treated 5, 10 or 30mM KSEB 14–01, and only in the 30mM propofol treated group (Fig. 5B).

## DISCUSSION

One mechanism by which general anesthetics are thought to work is via potentiation of the heteropentameric GABAAR through drug-specific binding sites. There are estimated to be 19 different subunit types creating a myriad of subunit stoichiometries, only a few of which have well-delineated structural data. Our previous work demonstrated efficacy of a new chemical class as both an anesthetic and an antiepileptic. Unlike propofol, these compounds produce minimal effects on breathing and hemodynamics in rats. They lack the free imidazole nitrogen of etomidate and therefore do not affect adrenal function. Both in silico and in vitro analyses show that their action is highly specific for a particular binding site within the GABA receptor located between subunits in the outer third of the transmembrane domain. Gamma-aminobutyric acid is an inhibitory neurotransmitter that has also been identified in mitochondria. A recent study [11] reported that GABAAR agonists can inhibit ischemic cell death in the brain and maintain mitochondrial membrane potential. These in vivo events have been considered likely secondary to reduced metabolic demand [11]. The results of the present in vitro study demonstrate in both neurons and astrocytes that either propofol or KSEB can preserve mitochondrial membrane potential, suggesting that a directly protective effect that originates within the mitochondria could also account for in vivo observations of protection with GABAAR agonists [11]. Interestingly, astrocytes, like neurons, express GABAARs, and prior work has identified GABAergic crosstalk between neurons and astrocytes elicited by anesthetic drugs [12]. Additional mechanistic studies are warranted to identify the mechanistic mitochondrial targets responsible for the protective effect of GABAAR agonist anesthetics.

During the normal process of oxidative phosphorylation, mitochondria generate ROS through consumption and reduction of elemental oxygen as the terminal electron acceptor for ETC for ATP production [13]. The routine generation of ROS is buffered by endogenous antioxidant systems both in the cytosol and in mitochondria, and ROS has even been evolutionarily incorporated into normal cellular function as an intracellular signaling molecule [13]. However, bioenergetic uncoupling of O2 reduction from ATP generation during injury or stress generates excessive ROS that overwhelm endogenous antioxidants, which can then lead to downstream lipid peroxidation, cellular dysfunction, and ultimately cell death [14]. Therefore, rates of ROS production often serve as a proxy for mitochondrial dysfunction. To generate electron flux through the ETC, mitochondria must also maintain the intracellular potential electrochemical gradient (mitochondrial membrane potential). Any reduction in the mitochondrial membrane potential will reduce electromotive force, decrease the reduction of O2, and ultimately reduce ATP production [15]. For this reason, measures of mitochondrial membrane potential represent a parallel indicator of mitochondrial dysfunction to rates of ROS generation. In the present study, both 10 and 30mM propofol and 10 and 30mM KSEB 14–01 treatment exhibited an antioxidant effect in neurons, which paralleled the protective effect observed in assays of mitochondrial membrane potential. However, this was not observed in astrocytes. This could be due to a lower rate of phototoxic ROS generation in astrocytes relative to neurons. This is supported by prior studies identifying that disruptions in mitochondrial membrane potential precede excessive ROS generation [15].

Another notable observation from the present study is preservation of OCR during substrate limitation with KSEB 14–01 and with the highest dose of propofol. In neurons subject to GD, OCR may be maintained or increased secondary to the extracellular acidification rate (ECAR) [16]. One limitation of the present study is that technical constraints with the Resipher system allowed us only to measure OCR in astrocytes and not in primary neuronal cultures. Astrocytes preferentially use glycolysis to generate ATP versus neurons, which utilize oxidative phosphorylation [17]. Our observations suggest that KSEB 14–01 may have reduced ATP demand to preserve overall energy supply, and thereby maintain respiration. Future investigations in neuronal cultures are warranted as the technology advances. However, the results of the present study suggest that KSEB 14–01 and higher doses of propofol may be useful for maintaining low metabolic states. Such a characteristic may prove potentially important in areas that require longer-term tissue preservation, such as various austere environments involving battlefield trauma, deep-sea operations, and long-term space travel.

Another limitation of the present study is the use of only mixed sex cell cultures, while there are known sex differences in mitochondrial function [18]. Prior work suggests [19, 20] that there are sex differences in how anesthetics affect mitochondria, with females often showing a greater resistance to anesthetic-induced mitochondrial dysfunction compared to males. This has been proposed as potentially due to influences of estrogen [20]. Whether there are sex differences in the direct effects of anesthetics on mitochondria, and whether there are age-related changes in the effects of propofol and our new class of selective GABAAR on mitochondrial function, is unknown and warrants future investigations.

In conclusion, using live-cell fluorescent assays in mouse brain cell cultures with intact mitochondrial reticula, we demonstrated that KSEB 14–01 appears to preserve both neuronal and astrocyte mitochondrial function in a dose-dependent manner, qualitatively similar in effect to propofol, but with greater effect. Such mitochondrial preservation characteristics, without the associated detriment in breathing and hemodynamics, continue to favor the development of this new agent and further emphasize the value of such mitochondrial-based analyses. Future in vivo work is warranted to explore the mechanistic targets on mitochondrial function involved in the anesthetic effects of both propofol and KSEB 14–01, especially for future applications.

## Supplementary Material

Supplementary Files

This is a list of supplementary files associated with this preprint. Click to download.

- FigureS1.tiff
- FigureS2.tiff

## Data Availability

all raw and processed data will be available upon request
